# Resource Availability Modulates Gene Expression Across Life Stages in a Migratory Butterfly

**DOI:** 10.1111/mec.70293

**Published:** 2026-03-08

**Authors:** D. Shipilina, L. Höök, K. Näsvall, V. Talla, A. Palahí, E. Parkes, R. Vila, G. Talavera, N. Backström

**Affiliations:** ^1^ Evolutionary Biology Program, Department of Ecology and Genetics (IEG) Uppsala University Uppsala Sweden; ^2^ Wellcome Sanger Institute Hinxton UK; ^3^ Institut de Biologia Evolutiva (CSIC‐Univ. Pompeu Fabra) Barcelona Spain; ^4^ Institut Botànic de Barcelona (IBB), CSIC‐CMCNB Barcelona Spain

**Keywords:** butterfly migration, environmental cues, gene expression, migratory syndrome, *Vanessa cardui*

## Abstract

Natural populations are in constant need of balancing resource allocation to compensate for seasonal environmental variation. In many insects, a well‐established trade‐off between migration and reproduction exists. While this trade‐off has been characterised phenotypically for decades, the underlying regulatory pathways are poorly understood. Here, we examined how resource‐related environmental cues shape transcription across development in the long‐distance migrant butterfly *Vanessa cardui* . In a multi‐cue, developmental stage‐specific design, adult females were exposed to host‐plant presence or absence, while larvae experienced food limitation or crowding. Adult exposure to host plants was associated with differential expression in ecdysteroid and juvenile‐hormone pathways, consistent with endocrine regulation of reproductive readiness and predictions of the oogenesis–flight syndrome. Larval resource limitation altered developmental and metabolic pathways, suggesting molecular predispositions and potential carry‐over effects to adult traits. Across all contrasts, metabolism emerged as a shared axis linking responses across life stages. Together, our results show that resource‐driven cues leave both stage‐specific and general transcriptional signatures that connect environmental context with the molecular basis of migratory behaviour.

## 1 Introduction

As animals adapt to the environment, they recurrently face challenges to optimise resource allocation and individual decisions can have considerable downstream consequences on both survival and reproductive output (Bell 2023; Stearns 1989). Migration is a complex biological adaptation that integrates behavioural, physiological and genetic traits and allows migratory organisms to avoid temporary unfavourable environmental conditions (Aidley 1981; Williams 1930, 1958). In insects, key cues that signal seasonal change include temperature, photoperiod, and variation in resource availability (Bauer et al. 2011; Gatehouse 1994). Resource‐limiting environmental conditions, such as fluctuations in host plant and nectar availability, influence life‐history traits across developmental stages and are likely to affect migratory behaviour (Denno et al. 1985; Johnson 1963). Although environmental cues are central for triggering migratory behaviour, the molecular mechanisms that underlie their perception and processing have been examined in detail in only a few migratory insects, most notably the locust *Locusta migratoria* (Kang et al. 2004) and the monarch butterfly *Danaus plexippus* (Zhan et al. 2011; Freedman and Kronforst 2023).

Accurate perception of environmental cues is key for the activation of a suite of synchronised behavioural, morphological and physiological traits essential for migration (i.e., the ‘migratory syndrome’), both in adult individuals and during ontogenesis (Angelo and Slansky Jr. 1984; Ramenofsky and Wingfield 2007). Yet responses can be specific for certain developmental stages: adults must for example often make rapid decisions on movement, while early developmental stages can encode longer‐term predispositions that shape later movement and reproductive strategies (Jiang et al. 2011). In long‐distance migrants, perception of host plants can mark the final phase of migration and dispersal, trigger onset of mating (Stefanescu et al. 2021) and a switch to reproductive investment. This perspective places host‐plant availability within the framework of the oogenesis–flight syndrome. This classic trade‐off refers to the delayed investment in reproduction in favour of migration (Johnson 1963; Rankin et al. 1986). Across taxa, expression of this syndrome varies: from complete reproductive arrest during migration (boll weevil; beet webworm) (Rankin et al. 1994; Cheng et al. 2012), to generation‐specific expression (monarch) (Malcolm et al. 2018), to little or no reproductive arrest (beet armyworm; codling moth) (Han et al. 2008; Gu et al. 2006), with some females even flying while carrying fertilised eggs (reviewed in Tigreros and Davidowitz 2019). This variation has been linked to nuanced responses to environmental conditions, as well as differences in hormonal regulation and life‐history strategies (Tigreros and Davidowitz 2019). It has been hypothesised that such cues modulate the strength of this trade‐off, yet the molecular pathways remain largely unexplored.

While host‐plant perception may act as a cue for adults to invest in reproduction, resource availability during development can shape migratory and reproductive strategies long before adulthood. Limited food availability and/or quality during development predominantly manifest in reduced body size, fat storage, fecundity, and delayed investment in reproduction (Boggs and Freeman 2005; Niitepõld 2019; Chen and Ruberson 2008). This effect can be further strengthened by competition arising from crowding, which we consider a form of competition‐driven resource limitation. A higher larval density can lead to a predominant investment in migration, likely as a strategy to disperse from areas where competition with conspecifics is high (Bauerfeind and Fischer 2005). The desert locust (*Schistocerca gregaria*) is a notable example of this phenomenon, exhibiting a density‐dependent phase polyphenism that triggers a transition from a benign, solitary phase to a more gregarious, highly migratory phase (Kang et al. 2004; Butler and Innes 1936). In Lepidoptera, density‐dependent migration has also been observed in the fall armyworm *Spodoptera frugiperda* (Wang et al. 2023), and larval density has been associated with outbreaks in the agricultural pest, beet webworm *Loxostege sticticalis* (Cheng et al. 2012). Taken together, the interplay between resource‐driven environmental cues across developmental stages underscores the need for a multi‐cue, multi‐stage approach. While most existing work has focused on phenotypic outcomes, the underlying gene‐expression responses to such resource limitation remain virtually unexplored.

The painted lady butterfly ( *Vanessa cardui* ) is an emerging model species for studying the genomic basis of multigenerational long‐distance migration (Reich et al. 2023; García‐Berro et al. 2023, 2025). This extraordinary migrant requires fine‐tuned regulation of resource allocation and environmental responsiveness at multiple developmental stages, combining rapid adult adjustments with long‐term developmental predisposition. Ease of rearing and recently developed genomic resources (Shipilina et al. 2022; Lohse et al. 2021) have now made it possible to investigate the genetic underpinnings of response to environmental cues. Several transcriptomic studies on *V. cardui* have explored larval host use (Celorio‐Mancera et al. 2016) and wing development (Connahs et al. 2016; Reed et al. 2020), whereas transcriptomic responses to resource‐limiting environmental cues remain largely unexplored. Spearheading work using chromatin accessibility and methylation profiling have pinpointed candidate pathways that can be involved in sensory perception of environmental cues and associations between epigenetic changes and expression levels (Boman et al. 2023), but analyses that investigate potential associations with transcription profiles of specific genes or gene categories have not been performed. Delayed onset of reproduction suggestive of an oogenesis‐flight syndrome has been observed in *V. cardui* (Stefanescu et al. 2021), but evidence is mixed (Wiklund and Friberg 2022) and considerable inter‐individual differences in migration distance and propensity have been observed (Reich et al. 2024, 2025). Host‐plant availability has been linked to reproductive activity (Näsvall et al. 2023), supporting interpretation of this environmental factor as a potential migratory cue. By combining well‐characterised migration ecology data with emerging genomic tools, *V. cardui* provides an ideal model for linking resource‐dependent environmental cues to the molecular mechanisms underlying life‐history trade‐offs in long‐distance migrants.

Here, we use the long‐distance migrant *V. cardui* as a model to link resource‐related environmental cues to gene expression trajectories across developmental stages and in adult females. We aim to identify molecular pathways shaping the trade‐offs faced by long‐distance migrants. Specifically, we test whether host plant availability induces reproductive investment detectable in gene‐expression patterns and whether these patterns align with predictions of the oogenesis–flight syndrome. We further test how resource limitation is integrated during development, revealing when and how early predispositions arise. To address these questions, we expose butterflies to environments that differ in host plant availability for egg laying (adult females), crowding and food limitation (across developmental stages). The results contribute to a deeper understanding of how environmental cues shape the molecular pathways underlying complex behaviours, such as long‐distance migration.

## 2 Methods

### 2.1 Experimental Setup

**FIGURE 1:**
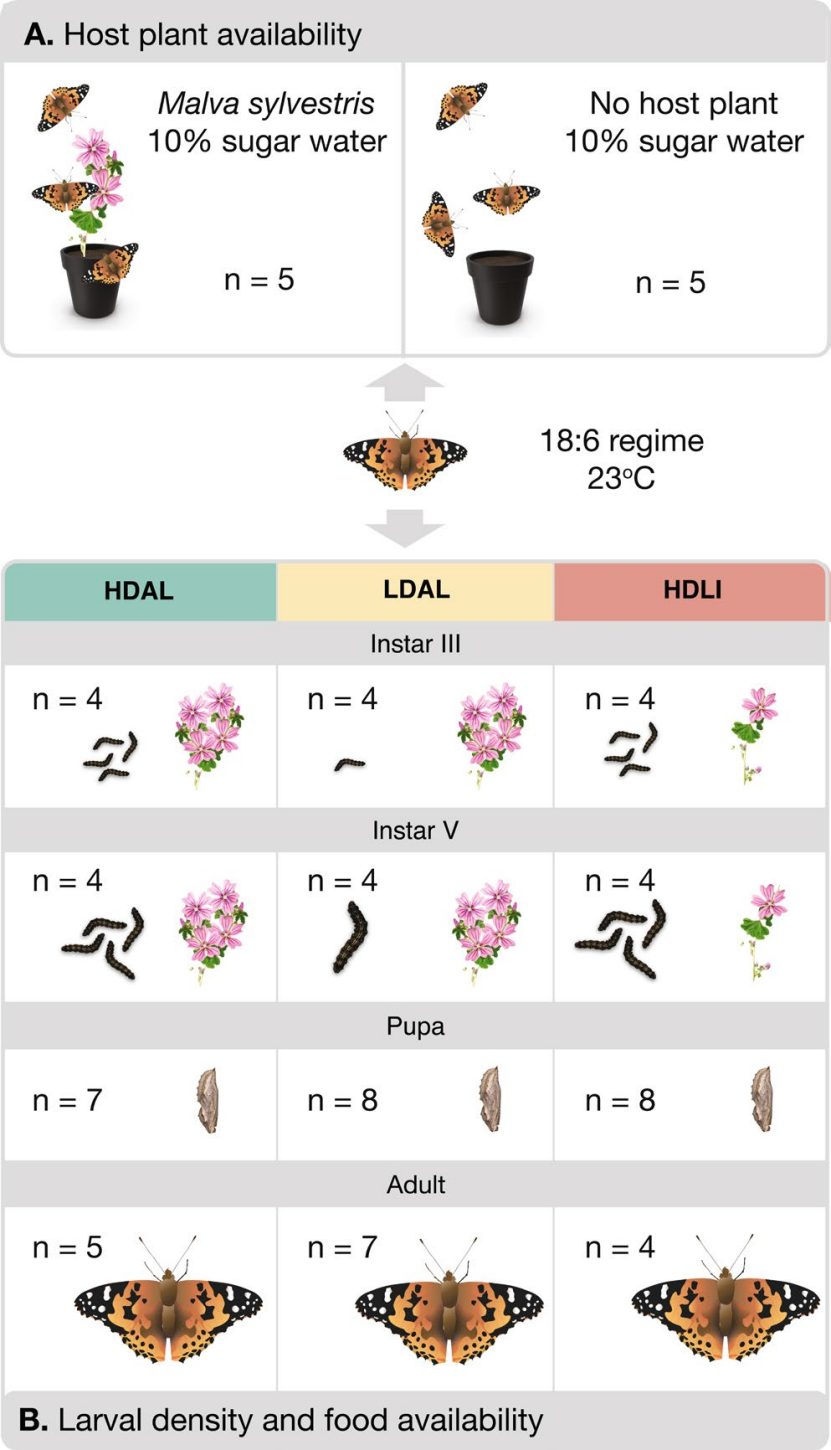
Setup of the two experiments conducted on offspring of wild‐caught *Vanessa cardui* females. Numbers of sequenced individuals are provided for each treatment and cohort. (A) The host plant availability experiment, where recently emerged females were divided in two experimental groups with or without access to *Malva sylvestris* for egg laying. (B) The setup of the larval density and food availability experiment for different developmental stages. Here, F2 offspring from five different F1 females were divided into three cohorts where the environmental conditions varied. HDAL = high density (10 larvae/flask) and *ad libitum* food, HDLI = high density (1 larva/flask) and limited food (fed every other day), LDAL = low density (1 larva/flask) and *ad libitum* food.

Painted lady (*Vanessa cardui*) butterfly females were collected in Catalonia, Spain, and individually housed in cages for egg laying at 23°C under an 18:6‐h light:dark regime. The butterflies were provided with host plants ( *Malva sylvestris* ) for egg laying and a 10% sugar water solution as a food source. The F1 offspring were raised individually with *ad libitum* access to food plants ( *M. sylvestris* ) and were subsequently divided into experimental groups under controlled environmental conditions (Figure 1).

Two separate experiments were carried out to analyse the transcriptomic response to different environmental cues. The first experiment was designed to investigate the potential influence of the presence or absence of the host plants for egg laying on gene expression profiles in adult females (Figure 1B). The second experiment was designed to characterise the potential effects of larval density and food availability on gene expression profiles during development (larval instars III and V, and pupae) and after emergence (imagines, both sexes) (Figure 1B).

### 2.2 Host Plant Availability Effects on Gene Expression Profiles in Adult Females

Twenty newly emerged adult F1 females were marked individually and released into one of two large cages (80 × 80 × 50 cm), 10 in each cage. One cage contained an abundance of host plants (9 15 × 15 cm pots with *M. sylvestris* ) for egg laying, while the other cage lacked host plants (Figure 1A) and both cages contained 10 free‐flying males. Both experimental groups were provided with 10% sugar water as a food source, and the temperature and light regime was the same as for rearing larvae (23°C and an 18:6‐h light–dark regime). In the morning 5 days after emergence, around the expected time for first mating/reproductive maturity (Stefanescu et al. 2021; Wiklund and Friberg 2022), five females from each respective treatment group were collected and snap frozen in liquid nitrogen.

### 2.3 Crowding and Food Availability Effects on Expression Profiles Across Developmental Stages

In the second experiment, five newly mated F1 females were placed in individual cages containing *M. sylvestris* for egg laying. The eggs (F2) laid by each female were collected and divided into three treatment groups (Figure 1): LDAL (low density, *ad libitum* food), HDAL (high density, *ad libitum* food), and HDLI (high density, limited food) (Figure 1B). In the LD (low‐density) condition, larvae were individually reared in 1‐l flasks, while in the HD (high‐density) treatment, 10 larvae were kept together in a single 1‐l flask per family. Both density treatment groups had *ad libitum* access to food ( *M. sylvestris* ), which was replaced daily. In the LI (limited resource) treatment group, the food was replaced every other day, creating a mild starvation regime. Individuals in this treatment group were again reared in groups of 10 (high density) in a single flask for each family. This set‐up allowed us to contrast treatments with different food availability (HDAL vs. HDLI) and larval rearing densities (HDAL vs. LDAL) separately (Figure 1).

Samples were collected at four developmental stages: larva (instar III and instar V), pupa, and adult. Larvae were harvested on the day they entered the respective larval stage, pupae were collected 1 day after pupation, and adults were harvested in the morning on the day of emergence. Prior to RNA extraction, individuals were snap frozen in liquid nitrogen and stored in −80°C. Note that larvae of this species cannot be easily sexed, and these cohorts therefore can constitute a mix of males and females. Pupae and adults, however, were sexed based on sex‐specific morphological characters (presence (female) or absence (male) of a suture on the 8th body segment in pupae and presence (♀) or absence (♂) of ‘claws’ on the front legs in imagines, respectively) and divided into sex‐specific cohorts. For each treatment and cohort, one individual among the offspring of each of the five different F1 females was selected for sequencing (Figure 1).

### 2.4 RNA Extraction and Sequencing

Two types of tissues were used for RNA extractions; heads (including antennae in adults, only head in larvae, top segment of the pupae) and abdomens (the 6th–8th body segments for all life stages). The number of samples for each treatment/cohort are provided in Figure 1 and Table S1. Tissues were homogenised using a micro‐pestle in guanidine‐isothiocyanate lysis buffer, followed by mixing with QiaShredder (Qiagen). RNA extractions were performed using the RNeasy Mini Kit (Qiagen) following the recommended guidelines by the manufacturer. RNA integrity and fragment lengths were assessed using 1% agarose gel electrophoresis, followed by measurements of the concentration using NanoDrop (ThermoFisher) and Qubit (ThermoFisher). Sequencing libraries were prepared using the Illumina TruSeq Stranded mRNA polyA selection kit and sequenced by the National Genomics Infrastructure (NGI) in Stockholm. Sequencing was conducted on two lanes of one S4 flow cell on the NovaSeq S6000 platform, generating 150 bp paired‐end reads.

### 2.5 Differential Expression Analysis

For all steps of the read processing, from adapter filtering to read mapping and transcript quantification, the Nextflow nf‐core (Ewels et al. 2020) pipeline rnaseq v3.8.1 was applied (Harshil et al. 2023). In brief, raw sequencing reads were trimmed using Cutadapt v.3.4 (Martin 2011) as implemented in Trim Galore! v0.6.7. STAR v2.7.10a (Dobin et al. 2013) was used for mapping the reads to a previously published genome assembly (Lohse et al. 2021). Read quantification was carried out using salmon v1.5.2 (Patro et al. 2017) and exported for future analysis as a DESeq2 object. Differential expression analyses were conducted in R v4.2.1 using DESeq2 v1.28.0 (Love et al. 2014).

To assess differential expression between the cohorts of adult individuals with or without access to host plants for egg laying, we employed the Wald test in the DESeq2. Our experimental design incorporated the correction for potential family effects, with treatment as the primary variable (~family + treatment). Due to incomplete family assignment for some samples, we utilised PCA analysis to recover the missing assignments. We applied the same Wald test for differential expression analysis in adult individuals subjected to environmental stressors: food limitation and larval crowding. Here, we also accounted for the potential effect of sex since both males and females were used in the analysis (~family + sex + treatment).

Differential gene expression across developmental stages was assessed using the likelihood ratio test mode of DESeq2 (model = ‘LRT’), which allows for analysis of time course experiments. This test compared the fit of a full model (~family+devstage+treatment+treatment:devstage) with a reduced model that excluded the interactive effect between the treatment and developmental stage (‘devstage’) variables. This analysis aimed to evaluate whether the effect of the treatment on gene expression differed across developmental stages. The same model was applied to both head and abdomen tissues, and the analysis was performed for the food availability (HDAL vs. HDLI) and rearing density (HDAL vs. LDAL) contrasts, respectively.

For further analysis, candidate genes were selected based on the criteria of an adjusted *p*‐value < 0.05 (Benjamini and Hochberg to control FDR) and a log fold change > 2. The GeneOverlap package (Li Shen 2017) was used to assess the significance of overlaps between candidate gene sets in different tissues. Since tests using LRT typically result in larger gene sets, clusterProfiler v3.17 (Wu et al. 2021) was applied to identify functional clusters within all sets of candidate genes across the four ontogenetic stages, using the parameters consensusCluster = TRUE and groupDifference = 2 on rlog‐transformed data. Regularised log (rlog) transformation was used for stabilising variance and normalising the count data. In the case of the head tissue, where 745 candidate genes were identified, more stringent clustering parameters were employed (groupDifference = 3). Functional information for differentially expressed genes was collected both by using previous annotations (Shipilina et al. 2022; Lohse et al. 2021) and by BLAST searches against the entire NCBI nucleotide database (Altschul et al. 1990). Data was summarised in corresponding Supporting Information tables (Tables S2, S3, S5, S7 and S10); when two annotations were available the one from the most similar ortholog was chosen.

To assess if certain functional categories were overrepresented in the gene‐sets with significant differential expression, we conducted two types of enrichment analyses, Gene Ontology (GO) terms and KEGG pathways, utilising previously obtained functional annotations. Enrichment analysis of GO terms (biological processes category) was performed using the TopGO package (Adrian Alexa 2017), employing the ‘weight01’ algorithm, with a significance threshold of *p* < 0.01. The enrichment analysis of KEGG terms was performed using the enricher module of clusterProfiler (Wu et al. 2021). All visualisations for this and the analyses described above were performed using the R package ggplot2 (Wickham 2016). For plots where it was not possible to display all gene names, we used the geom_text_repel() function to label a random subset of genes for visualisation.

## 3 Results

### 3.1 Gene Expression Patterns in Response to Host Plant Availability in Adult Females

**FIGURE 2:**
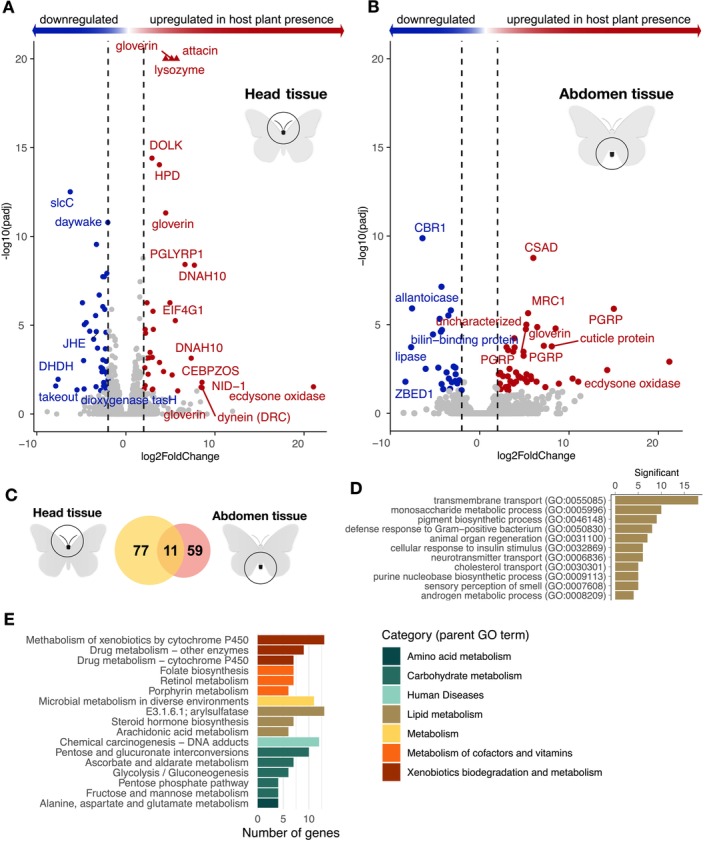
Volcano plots illustrating the relative levels of gene expression in the head (A) and abdomen (B) of adult females (*x*‐axis; log2 fold change) in the two treatment groups with and without access to host plants for egg laying. Genes with a fold change difference > |2| and FDR‐adjusted *p*‐value < 0.05 are depicted in red (significantly higher expression in the treatment with host plants) and blue (significantly higher expression in the treatment without host plants), respectively, while non‐significant genes are illustrated by grey dots. Only selected outlier gene names are shown. (C) Venn diagram showing the number of overlapping and unique differentially expressed genes between the two tissues (head = orange, abdomen = yellow). (D) Bar plot showing the counts of the enriched (FDR‐adjusted *p* < 0.01) gene ontologies for the significantly differentially expressed genes between the treatment groups for both tissues combined. (E) Bar plot illustrating the numbers of significantly differentially expressed genes enriched for KEGG pathways, both tissues combined. The higher hierarchical grouping is indicated by bar colours (legend to the right).

To understand how the presence or absence of host plants for egg laying affects transcriptional responses in recently emerged female imagines, we analysed gene expression in head and abdominal tissues (Figure 2). The rationale behind choosing those particular tissues was that signalling cascades should be initiated in the head based on sensory perception of the environmental cues and that this may manifest in temporal differences in investment in reproduction and migration, which might be picked up by gene expression differences in the abdomen (where the gonads are located). In total, abdominal tissue was analysed in 10 individuals (five for each treatment) and head tissue in 9 individuals (five and four individuals from the treatments with and without access to host plants for egg laying, respectively). In the head, we found that 88 genes were significantly differentially expressed (*p* < 0.05 after FDR adjustment) between treatment groups. Of those, 34 genes (0.3% of all genes analysed) had higher and 44 had lower expression (0.4%) in the treatment with access to host plants compared to the treatment without host plants (Figure 2A). In the abdomen, the corresponding numbers were 70 differentially expressed genes; 44 (0.4%) with higher and 26 (0.2%) with lower expression in the treatment with access to host plants (Figure 2B).

We found that significantly differentially expressed genes encompassed a diverse range of functional categories (Figure 2A,B), including immune genes (gloverin, attacin, PGRP), metabolic genes (lipase), and genes involved in endoskeleton formation (cuticle protein) (Figure 2, Table S3). Of particular interest was the ecdysone oxidase gene, which exhibited a remarkable upregulation in both head and abdomen in the individuals that had access to host plants for egg laying (Figure 2A,B; Note that DESeq2 may sometimes exaggerate outliers—Li et al. 2022). A list of the top significantly differentially expressed genes (*p* < 0.01) and their putative functions is provided in Table S2.

Among the genes that were differentially expressed between the adult female treatment groups, 11 genes were found in both tissues (Figure 2C). To gain further insights into the associations between differentially expressed genes and functional categories and increase statistical power, we combined the results from both tissues and assessed if any GO‐terms were enriched. Significantly overrepresented GO‐terms encompassed transmembrane transport, various metabolic processes (including ecdysone biosynthesis), and defense response (Figure 2D, Table S3). Consistent with this finding, the analysis of overrepresented KEGG pathways revealed enrichment of different metabolic pathways, in particular lipid, carbohydrate, vitamin, and xenobiotic metabolism (Figure 2E, Table S4).

### 3.2 Gene Expression Variation Associated With Food Availability During Development

**FIGURE 3:**
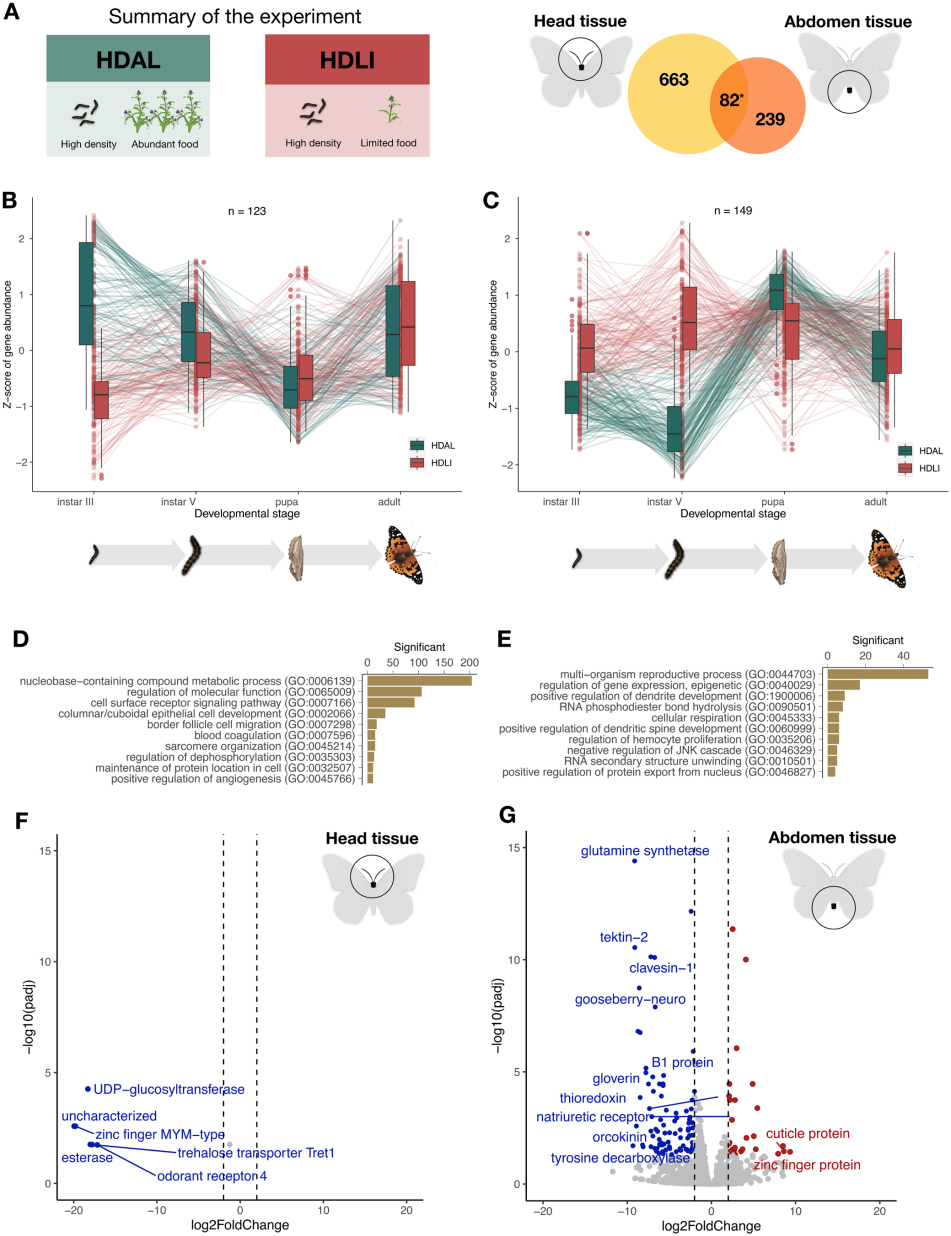
A summary of the results from the comparison between experimental groups with access to different amounts of food plants during development. (A) Summary of the experiment and a Venn diagram showing the number of differentially expressed genes in each respective tissue (left = head, right = abdomen) and the number of genes differentially expressed in both tissues (centre). The star indicates that the number of overlapping genes was significantly higher than expected by chance. (B, C) Box plots showing the temporal patterns of differential expression across ontogenetic stages in head (B) and abdomen (C). Outliers are indicated with circles, temporal trends of gene expression levels for specific genes are illustrated with lines. (D, E) The top 10 most significantly overrepresented GO terms (*p* < 0.01) for differentially expressed genes in head (D) and abdomen (E). (F, G) Volcano plots showing the relative levels of gene expression in the adult individuals for head (F) and abdomen (G). Genes that are significantly differentially expressed and meet the threshold (FDR‐adjusted *p*‐value < 0.05), log fold change difference < |2| are highlighted. Red marks genes that are upregulated in adults in the food limitation treatment (HDLI), while blue mark genes that are upregulated in the treatment where larvae had access to unlimited food (HDAL).

To complement the analysis in adult females, we focused on investigating differential gene expression across developmental stages in experimental cohorts exposed to environments that varied in food availability and rearing density. Again, we focused on the head and abdomen for the same reasons as indicated above. For the contrast between experimental groups with differences in food availability during development (HDAL vs. HDLI), the likelihood ratio tests revealed 745 and 321 significantly differentially expressed genes (FDR‐adjusted *p*‐value < 0.05) in the head and abdomen, respectively. Notably, the two sets of genes with differential expression in the two respective tissues demonstrated a high degree of overlap (Jaccard index = 0.1, *p*‐value = 9.6 × 10^−30^; Figure 3A). To check which stages contributed the most to the overall differences in expression patterns between treatment groups, we performed a clustering analysis which groups genes based on the expression patterns across developmental stages, facilitating the identification of genes with similar profiles and potential functional relationships. In head tissue, the most prominent cluster comprised 123 genes (16.5% of the differentially expressed genes in this tissue; Figure 3B). The majority of expression differences within this cluster were observed in instar III larvae. Similarly, in the abdominal tissue, 149 genes (46.4% of the differentially expressed genes) formed a distinct cluster. Genes within this cluster predominantly showed differential expression in instar V larvae (Figure 3C).

The GO term analysis for differentially expressed genes in head tissue revealed both a general enrichment of functions related to metabolic processes and regulation, and more specifically also enrichment of functions associated with epithelial cell development, sarcomere organisation, angiogenesis regulation and blood coagulation (Figure 3D, Table S5). Differentially expressed genes in abdominal tissue were predominantly associated with reproductive processes, and neural and immune cell development (Figure 3E, Table S6). In addition, a joint KEGG pathway analysis of both tissues unveiled that functions associated with ribosome biogenesis (ko03008) and aflatoxin biosynthesis (ko00254) were overrepresented.

The expression trajectories across developmental stages show that the influence of the environmental factors on gene expression differences between experimental groups in general appears to diminish at the pupal and adult stages. In order to investigate how environmental cues experienced during development are manifested in recently emerged imagines in more detail, we compared differences in gene expression between adult individuals that had experienced different environmental conditions during development specifically (Figure 3F,G). In head tissue, only six genes (*p* < 0.05) showed significantly differential expression: *Tret1, odorant receptor, UDP‐glucosyltransferase, esterase and zinc‐finger MYM* (Table S7). These genes were downregulated in response to limited food treatment. In the abdominal tissue, 189 (*p* < 0.05) genes were found to be differentially expressed between treatment groups. Among the most prominent outliers were *cuticle protein* (upregulated in response to limited food source), *gloverin, glutamine synthetase, tektin, clavesin, gooseberry‐neuro, orcokinin* and *tyrosine* (downregulated in response to limited food source) (Table S7).

### 3.3 Gene Expression Variation Associated With Rearing Density During Development

**FIGURE 4:**
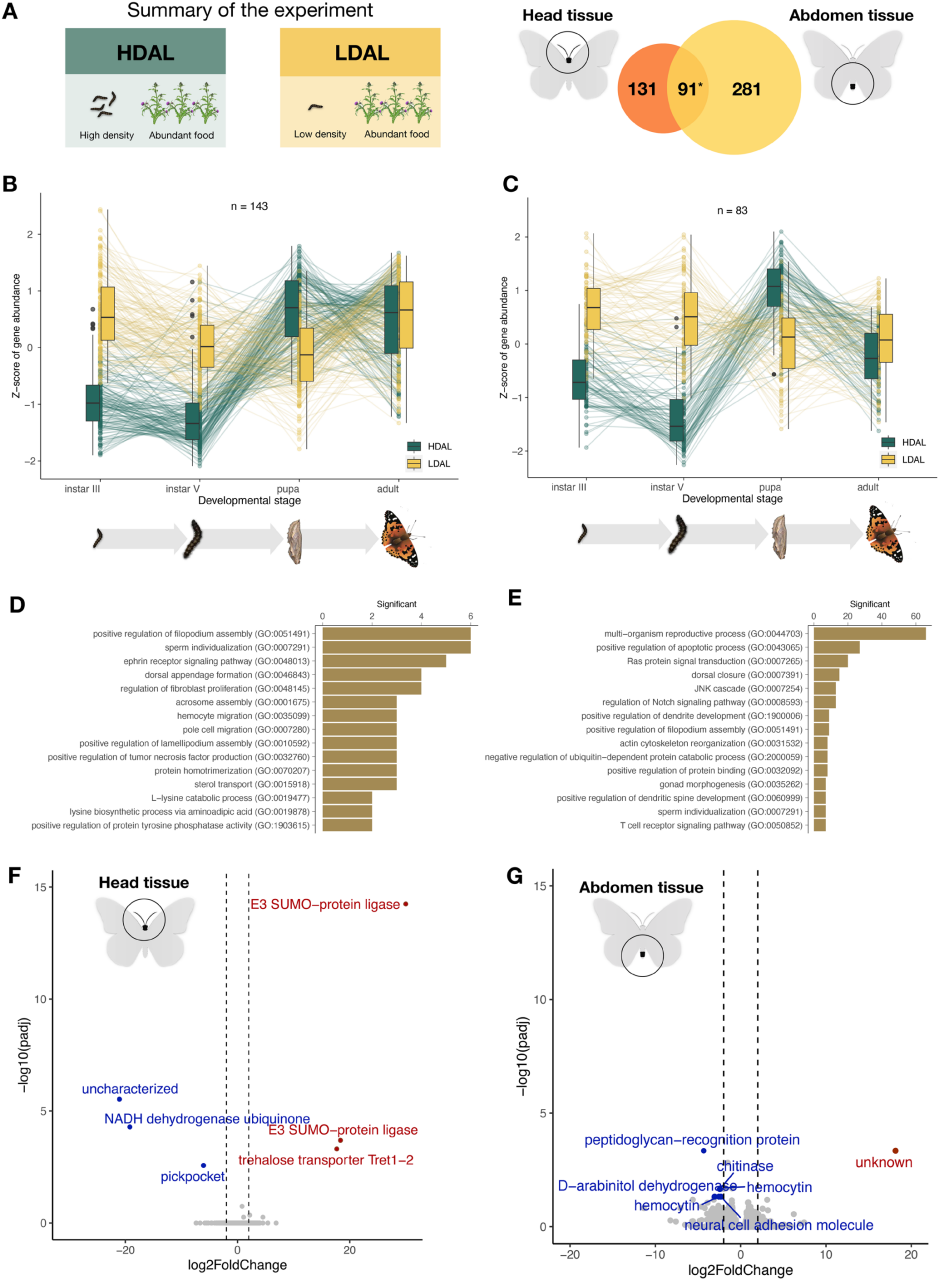
A summary of the results from the comparison between experimental groups, which were reared at different densities during development. (A) A Venn diagram showing the number of differentially expressed genes in each respective tissue (left = head, right = abdomen) and the number of genes with significantly different expression in both tissues (centre). The star indicates that the number of overlapping genes was significantly higher than expected by chance. (B, C) Box plots showing the temporal patterns of differential expression across ontogenetic stages in head (B) and abdomen (C). Outliers are indicated with circles, temporal trends of gene expression levels for specific genes are illustrated with lines. (D, E) The top 10 most significantly overrepresented GO terms (*p* < 0.01) for differentially expressed genes in head (D) and abdomen (E), respectively. (F, G) Volcano plots showing the relative levels of gene expression in the adult female individuals for head (F) and abdomen (G). Genes that are significantly differentially expressed and meet the threshold (FDR‐adjusted *p*‐value < 0.05), log fold change difference < |2| are highlighted. Red marks genes that are upregulated in adults in low density treatment (LDAL), while blue marks genes that are upregulated in the treatment where larvae had access to unlimited food (HDAL).

To complement the analysis of gene expression variation associated with food plant availability during development, we also compared treatment groups that were reared at different densities (10 larvae vs. 1 larva per flask, HDAL vs. LDAL). In this comparison, we found a large number of genes differentially expressed in both the head (222 genes) and abdomen (372). There was also a significant overlap between the tissues, that is, a higher proportion of genes were differentially expressed in both tissues than expected by chance (Jaccard Index = 0.2, *p*‐value = 1.2 × 10^−80^; Figure 4A).

To investigate the temporal trajectories of differences in expression patterns between treatment groups during development, we again performed a clustering analysis based on the expression patterns across the different developmental stages. In head tissue, the two main clusters contained 143 (38.4% of the differentially expressed genes in this tissue; Figure 4B) and 69 (18.5%; Figure S1) genes, respectively. Visual inspection clearly showed that expression differences in instar III and V larvae were driving the overall patterns within these clusters (Figure 4B). In the abdominal tissue, 83 genes (37.4% of the differentially expressed genes in this tissue) formed a distinct cluster (Figure 4C). Again, this cluster was mainly distinguished by considerable differences in gene expression in the larval stages (Figure 4C).

The gene ontology enrichment analysis of functional roles of differentially expressed genes in these clusters revealed that the most enriched functional category was regulation of filopodia assembly in head (Figure 4D). We also found enrichment of ontology terms related to sperm maturation, ephrin signalling, and several other functional categories (Figure 4D, Table S8). In the abdomen, there was a significant enrichment of GO terms associated with reproductive processes, including functions such as egg formation, egg laying, and mating development (Figure 4E, Table S9). In addition, there were several enriched terms associated with signal transduction like ephrin signalling, Ras signal transduction (involved in cell growth, division, and differentiation), Notch signalling (associated with neurogenesis), and JNK signalling (regulation of ubiquitin‐dependent processes).

Analogous to the analysis based on food plant availability, we compared differences in gene expression between recently emerged females that had experienced different levels of crowding during development (Figure 4F,G, Table S10). In this comparison, six genes showed significant differences in expression level in head tissue, of which functional information was available for five (Figure 4F). We found that two copies of the *SUMO ligase* and a *trehalose transporter* were significantly higher expressed in the low density (LDAL) compared to the high density treatment group (HDAL). The genes *pickpocket* and *NADH dehydrogenase*, in contrast, had a significantly higher expression in the HDAL than in the LDAL treatment group. In the abdominal tissue, the *peptidoglycan recognition protein (PGRP)*, *chitinase*, *hemocytin*, D‐*arabinitol*, and *NCAM* were significantly overexpressed in the LDAL treatment group compared to HDAL, while no genes with known functions had higher expression in HDAL.

## 4 Discussion

Insect migration is tightly linked to the perception and processing of seasonal environmental cues (Chapman et al. 2015; Dingle 2014). Among these cues, changes in resource availability, such as host‐plant abundance, shape insect life‐history strategies. Yet the molecular basis of these responses remains largely unexplored, either those triggered during adulthood or across developmental stages. Our study addresses this gap with a multi‐cue, multi‐stage transcriptomic design in the long distance migrant *V. cardui* . By analysing both adult females and different ontogenetic stages in experimental contrasts, we identify different responses to resource‐related environmental cues. First, we show that host plant abundance is strongly associated with differential expression of genes in the ecdysteroid and juvenile‐hormone pathways in adult *V. cardui* females. This pattern is consistent with endocrine modulation of reproductive readiness and aligns with predictions of the oogenesis–flight syndrome. Second, we show that larval starvation and crowding predominantly induce molecular signatures in developmental and metabolic processes, suggesting potential carry‐over effects on adult traits. These results support an association between environmental cues and the migratory syndrome in insects and motivate future experimental work to test the underlying mechanistic links. Metabolism also emerges as a shared axis across larval and adult stages. Below, we discuss these results in more detail.

### 4.1 The Response of Adult Females to Differences in Host Plant Availability

In adult *V. cardui* females, we found that access to host plants for oviposition predominantly is associated with shifts in the expression of hormone‐related genes, consistent with endocrine modulation of reproductive readiness. We detected significant changes in multiple regulators of developmental hormones, with ecdysone oxidase showing the largest difference in expression between treatment groups. Ecdysone is a steroid hormone crucial for numerous biological processes in metamorphic insects during major developmental transitions, including the maturation of oocytes and control of oviposition (Niwa and Niwa 2014; Robbins et al. 1968; Swevers and Iatrou 2003). Ecdysone oxidase in turn regulates the levels of active ecdysone by converting it to 3‐dehydroecdysone and vice versa, thereby controlling the availability of active hormones through a rapid feedback mechanism. We propose that increased expression of ecdysone oxidase, triggered by the availability of host plants, modulates these hormone levels, enhancing reproductive investment (Cheng et al. 2018). Additionally, we found that juvenile hormone (JH) pathway genes are downregulated in the absence of host plants in our experiment. This is evident from the expression profiles of genes such as juvenile hormone esterase (JHE) which regulates levels of juvenile hormone (Herman 1981; Doyle et al. 2022; Zhang et al. 2020) and *daywake* and *takeout* genes which encode juvenile hormone binding proteins. These results are consistent with a previous chromatin accessibility study highlighting the role of JHE in response to access to host plants for egg laying in *V. cardui* females (Näsvall et al. 2023) and strengthen the evidence for the importance of the JH pathway in regulating the migration–reproduction balance in insects in general. Importantly, the results also allow us to link host plant availability to the oogenesis–flight syndrome in *V. cardui* , corroborating data from field studies (Stefanescu et al. 2021).

It is also worth noting that several candidate genes identified in our experiment correspond to genes previously implicated in the oogenesis–flight trade‐off in other insect systems. Hormonal regulation cascades, such as the juvenile hormone (JH) pathway, significantly contribute to the reproduction‐migration trade‐off in monarch butterflies (Herman 1981; Green 2021). Additionally, genes involved in JH synthesis have been shown to be significantly overexpressed in migrating compared to sedentary hoverflies (Doyle et al. 2022). Although our data and approaches do not allow us to establish a causative association between host plant availability and investment in reproduction or migration per se, the gene expression analysis revealed a set of candidate genes that can be used to investigate the molecular underpinnings of the reproduction‐migration trade‐off in more detail. While these particular findings illuminate adult responses to environmental cues, our results from the analysis of different ontogenetic stages also point to earlier developmental processes that may shape these predispositions.

### 4.2 The Effects of Rearing Density and Food Availability on Gene Expression Patterns Across Developmental Stages

Adult behavioural decisions and environmental responsiveness may be shaped by conditions experienced earlier in development (Yoon et al. 2023). Both crowding and food resource availability have for example been shown to impact the timing of development and morphology of migratory insects, which in turn directly affect the flight response norms and migration propensity (Bhavanam and Trewick 2022; Zhang et al. 2019). Insects experiencing starvation in general exhibit delayed development (Chen and Ruberson 2008), prolonged larval stages and reduced body sizes (Zhang et al. 2019), and these effects have also previously been observed in *V. cardui* (Kelly and Debinski 1999). Similarly, negative associations between larval density and developmental rates have been established (Bauerfeind and Fischer 2005). This evidence arises from direct measurement of resulting phenotypic traits, while molecular mechanisms involved in the responses are less studied (but see e.g., Wang et al. 2023). In our study, transcriptomic signatures of the response to periodic starvation and larval crowding confirmed differences in activation of developmental genes and pathways in multiple organ systems. Differentially expressed genes were enriched for categories associated with epithelial cell and dendrite development, sarcomere organisation, angiogenesis, and hemocyte proliferation, indicating a connection between resource limitation and molecular alterations in developmental pathways. Furthermore, differential expression of genes involved in neural development and reproduction point to potential carry‐over effects of the larval environment on adult reproductive and migratory strategies in *V. cardui* , consistent with recent evidence of methylation changes in larvae under resource stress (Boman et al. 2023). Building on this, we analysed how gene expression varied across developmental stages in more detail.

Since the analysis spanned multiple developmental stages, from larval instar III to recently emerged imagines, we gained insights into the temporal variation in gene expression and identified critical developmental periods where the effect was particularly pronounced. Notably, the most significant gene expression differences occurred during the larval stages in both experiments, especially in instar V larvae—the final larval stage for *V. cardui* and a critical time point for responding to environmental cues before metamorphosis (Truman and Riddiford 2019). Our findings highlight genes associated with programmed cell death, such as E3‐type small ubiquitin‐like modifier (SUMO) (Enserink 2015), and show enrichment in the JNK and Ras signalling pathways, essential for the tissue remodelling required during metamorphosis (Tettamanti and Casartelli 2019). It should be noted that the last larval stage is accompanied by considerable physiological changes, and environmental shifts during this time period can therefore have particular importance for plastic responses later in life (Mirth et al. 2021).

Finally, the larval density experiment triggered differential expression of genes related to development of reproductive systems, which is illustrated by ontology terms such as sperm individualization and gonad morphogenesis. Early changes in reproductive functions are particularly noteworthy in the context of the trade‐off between reproduction and migration on the adult stage, a topic previously discussed in relation to adult experiments. These results underscore the role of the male reproductive system as well and are in line with observations in other lepidopterans, such as *Plodia interpunctella* and *Mythimna separata*, which have been shown to have increased sperm production in response to crowding (He and Miyata 1997). Previous experiments have revealed that a minor proportion of actively migrating painted lady females already are mated, and the variance in age at first mating is considerable (Stefanescu et al. 2021). In addition, although a single mating seems to be the most common, around 30% of 198 wild‐caught females had mated 2–3 times (Stefanescu et al. 2021). This indicates that mate competition can be strong—in particular if the density of conspecifics is high—and this could potentially foster investment in sperm production under certain conditions (e.g., if crowding is significant). This is of course quite speculative and more detailed analysis of the relationship between crowding and mate competition will be needed before robust conclusions can be made.

### 4.3 Host Plant Availability Affects Gene Expression Patterns Across All Life Stages

Bringing together responses from all life stages, our results suggest that resource limitation activates a shared physiological axis: alterations in metabolism at the molecular level appear to be a consistent response to the environmental cues across all developmental stages. In adults with or without host‐plant access, we observed differential expression of genes involved in carbohydrate and lipid metabolism. Under larval starvation, we identified over 200 differentially expressed genes associated with the ‘compound metabolic process’, including the trehalose transporter (*Tret1*) in adults that had experienced starvation during development. Trehalose is the primary sugar found in insect hemolymph, synthesised in the fat body and subsequently distributed by transporters (Kikawada et al. 2007). Moreover, metabolic regulation is central to the physiology of migratory insects, supporting sustained flight and reproductive investment. Thus, while larvae and adults employ these pathways for different immediate purposes, the recurrence of metabolic response across stages suggests broader, interconnected regulatory networks that coordinate how migratory insects integrate environmental information throughout ontogenetic stages.

Our results also indicate that the expression of immune response genes can be affected by environmental conditions. We found, for example, upregulation of several immune genes in adult females when no host plants were available. It should be noted that the role of immunity in the migratory syndrome is multifaceted. Energy may, for example, be allocated from immune functions to migration‐related traits, but immunity pathways may also be activated in response to the potentially more diverse pathogens encountered during migration (O'Connor et al. 2018; Shaw et al. 2023). Although we cannot establish causality between expression differences of immune genes and investment in reproduction or migration, immune gene evolution has been shown to be dynamic in migratory species in general (O'Connor et al. 2018) and may be of particular importance in *V. cardui* where several immune genes are uniquely present in multi‐copy arrays (Shipilina et al. 2022).

## 5 Conclusions and Future Directions

Here we provide insights into the complex responses to environmental cues in insects and relate them to the migratory syndrome in a migratory butterfly species. Signatures consistent with reallocation of resources were observed in the host plant experiment, which aimed to initiate the trade‐off between migration and reproduction. A key finding is the crucial role of hormonal regulation in this response. We examined the early predisposition for migratory plasticity by subjecting larvae to different environmental cues, such as food abundance and larval crowding. This experiment allowed us to closely examine the timing of the environmental cue perception and track this process throughout development. This study identifies candidate genes and pathways underlying transcriptomic responses to resource limitation, linking environmental cues to molecular and developmental processes and potentially oogenesis‐flight syndrome in a long‐distance migratory insect. Interpretation of the functional effects of candidate loci in this study is based on homology‐based functional annotation, which can suggest putative functions, but does not directly test them. Future work should therefore validate key candidates using direct approaches (e.g., targeted expression assays such as qPCR) with particular emphasis on genes in the ecdysone and juvenile hormone pathways, as well as candidates involved in trehalose transport and neural regulation. More broadly, our results describe associations between environmental conditions and adult physiological states relevant to migration in a butterfly and provide a set of candidates for testing causal functional links in future studies.

## Author Contributions

D.S. designed research, analysed data and wrote the first draft of the paper. L.H., K.N., V.T., A.P. and E.P. performed experimental research and contributed to data analyses. R.V. and G.T. provided conceptual support and contributed to data analysis and writing the final version of the paper. N.B. designed research, performed experimental research, provided support for the analyses and contributed to the writing of the first draft. All authors read and approved the final version of the article.

## Funding

This work was funded by a research grant from the Swedish Research Council FORMAS (grant # 2019‐00670 to N.B.) and the Swedish Collegium for Advanced Science (Natural Sciences Programme, Knut and Alice Wallenberg Foundation, Postdoc funding for D.S.). R.V. was supported by grants PID2022‐139689NB‐I00 (funded by MCIN/AEI/10.13039/501100011033 and ERDF, EU) and 2021‐SGR‐00420 (Departament de Recerca i Universitats, Generalitat de Catalunya). G.T. was supported by grants PID2023‐152239NB‐I00 MICIU/AEI/10.13039/501100011033 and the LINKA20399 from the CSIC iLink programme.

## Conflicts of Interest

The authors declare no conflicts of interest.

## Supporting information


**Data S1:** mec70293‐sup‐0001‐TableS1‐S10.xlsx.

## Data Availability

The data that support the findings of this study are openly available. RNA‐seq data are available at the European Nucleotide Archive under PRJEB107093. Scripts are available on GitHub in the following repository: https://github.com/EBC‐butterfly‐genomics‐team/EnvironmentalCues_RNA_Vanessa_cardui. Benefits from this research accrue from the sharing of our genomic data and results on public databases as described above.
